# Bacterial Community Characteristics of *Kengyilia thoroldiana* Rhizosphere Soil in Different Topographic Habitats of the Yellow River Source Region and Their Response to Vegetation-Soil Factors

**DOI:** 10.3390/microorganisms13112438

**Published:** 2025-10-24

**Authors:** Liangyu Lyu, Pei Gao, Yunfei Xing, Jun Ma, Yan Liu, Zhijie Yang, Xin Wang, Jianjun Shi

**Affiliations:** 1 Academy of Animal Husbandry and Veterinary Sciences/Key Laboratory of Adaptive Management of Alpine Grassland, Qinghai University, Xining 810016, China; yb230909000074@qhu.edu.cn (L.L.); y200954000466@qhu.edu.cn (P.G.); qhdxxyf@163.com (Y.X.); 13997471052@163.com (Y.L.); 15003600361@163.com (Z.Y.); 15110946395@163.com (X.W.); 2 Graduate School, Qinghai Minzu University, Xining 810016, China; 3 Grassland Improvement Experimental Station, Gonghe 813000, China; 18599055205@163.com

**Keywords:** topographic factors, Qinghai-Tibet plateau, FAPROTAX tool, rhizosphere microbial community, soil–vegetation interaction, soil pH

## Abstract

This study aims to uncover the structural and functional characteristics of rhizosphere soil bacterial communities of *Kengyilia thoroldiana* under five types of topographic habitats in the source region of the Yellow River, and to explore the interaction mechanisms between bacterial communities and plant-soil factors, thereby providing microbiological support for the ecological restoration of *Kengyilia thoroldiana* artificial grasslands in alpine desert grassland. In this study, high-throughput sequencing technology was employed to compare the species composition, diversity, interaction networks, and functional characteristics of rhizosphere bacterial communities of *Kengyilia thoroldiana* across five topographic habitats in the source region of the Yellow River. Additionally, Mantel tests and redundancy analysis (RDA)) were conducted to explore the key environmental factors driving the structure of bacterial communities. The results showed that habitat differences significantly influenced the community characteristics of *Kengyilia thoroldiana* and soil physicochemical properties. The plant height, coverage, biomass, and soil carbon, nitrogen, and phosphorus contents were highest in habitats H2 and H5, while they were lowest in habitats H1 and H3. In contrast, soil pH and electrical conductivity exhibited an opposite trend. At the bacterial community level, the number of operational taxonomic units (OTUs) in habitat H5 reached 1917, with α-diversity indices such as Shannon, Ace, and Chao1 being 6.13, 1820.85, and 1844.80, respectively, significantly higher than those in habitat H1. Cluster analysis revealed that habitat H3 formed a distinct group, while the bacterial community structures in the remaining four habitats were similar. Functional prediction indicated that chemoheterotrophy and aerobic chemoheterotrophy were the dominant functions across all habitats, with functional expression values exceeding 9300 in habitats H2, H4, and H5. Redundancy analysis confirmed that soil pH and SOC were the key factors driving the structure of rhizosphere bacterial communities of *Kengyilia thoroldiana*. In summary, topographic habitats influence the growth of *Kengyilia thoroldiana* plant communities by shaping soil environmental heterogeneity, thereby regulating the structure and function of rhizosphere bacteria associated with *Kengyilia thoroldiana*.

## 1. Introduction

*Kengyilia thoroldiana* is a perennial herb of the genus *Kengyilia* in the Poaceae, which grows in the alpine desert grassland, and the soil types are mainly alpine meadow soil and sandy soil [1]. It is mainly distributed across central Asian high-altitude regions, encompassing Qinghai, Tibet, southeastern Xinjiang, and northern Sichuan, where it grows on slopes, in river valleys, and in sandy environments at elevations ranging from 3300 to 5000 m [2,3]. As a native grass species in alpine desert grassland, *Kengyilia thoroldiana* features pendant or lateral rhizomes that effectively stabilize shifting sands and prevent desertification, making it a pivotal species for vegetation restoration and reconstruction in the Yellow River source basin [4].

The plant rhizosphere, as a specialized microenvironment where root systems interact with soil, represents the core arena for interactions among soil, roots, and bacteria. The essence of these interaction mechanisms lies in the multifaceted functional regulation by root exudates [5,6,7]. Root exudates inhibit the proliferation of pathogenic bacteria by secreting antimicrobial agents such as terpenoids, and trigger the regulation of gene expression in rhizobial bacteria through flavonoid chemical signaling molecules [5,6,7]. This process promotes the proliferation of beneficial bacteria while suppressing harmful bacteria, thereby optimizing the structure of the rhizosphere bacterial community. As the core driving force behind material transformation and nutrient cycling in soil ecosystems, rhizosphere soil bacteria deeply participate in key ecological processes such as organic matter decomposition, element cycling, and humus transformation, owing to their rich species diversity and high metabolic activity [8,9,10]. The specific manifestations are as follows: nitrogen-fixing bacteria provide nitrogen through nitrogen fixation, supporting seed germination and vegetative growth [8]; phosphate-solubilizing bacteria dissolve insoluble phosphorus by secreting organic acids, also supporting seed germination and vegetative growth [8]. Pseudomonas synthesize indole-3-acetic acid (IAA) to promote root elongation and lateral root formation [9]. Additionally, deaminases produced by certain bacteria can reduce plant ethylene levels, enhancing survival under abiotic stresses such as drought and salinity [10]. The functional roles of these rhizosphere bacteria directly contribute to improved plant nutrient uptake efficiency, enhanced growth and development, and increased stress resistance, forming an efficient ecological interaction network.

Species differences induce variations in genotype, root system architecture, and root exudates, directly leading to the differentiation in structure and function of rhizosphere bacteria [11]. Plant genotypes achieve the recruitment of specific functional bacteria by regulating root exudates, while simultaneously controlling the diffusion range of exudates and their contact efficiency with soil bacteria through root morphological traits such as root length and root hair density [11]. Among environmental factors, soil pH can regulate bacterial activity and community structure, nutrient gradients can determine bacterial competitive relationships, and water conditions can influence the concentration of bacterial exudates [12,13,14]. These intrinsic plant genotype differences and extrinsic environmental factors, through direct-indirect interactions, jointly govern the recruitment dynamics and community maintenance of rhizosphere bacteria. Ultimately, they form a synergistic interaction network encompassing nutrient exchange, disease resistance defense, and environmental adaptation, thereby optimizing the ecological functions of the plant–bacteria system [11,12,13,14].

In recent years, research on *Kengyilia thoroldiana* has predominantly focused on foundational areas such as plant morphological characteristics, stress resistance (mechanisms of cold and drought tolerance), and artificial introduction and domestication, with relevant studies still in their early stages [1,2,3,4]. A notable gap exists in current research—studies on the rhizosphere soil bacterial community structure of *Kengyilia thoroldiana* and its interactions with habitat factors are particularly scarce. To address this research gap, this study takes the rhizosphere soil of *Kengyilia thoroldiana* from five typical topographic habitats in the Yellow River source basin as the research object, based on the two core hypotheses that specific topographic habitats can directionally recruit functional microbial communities and that certain habitat factors such as soil and vegetation characteristics can significantly influence the differential evolution of rhizosphere bacterial community structures and further regulate their metabolic functions. By employing Illumina MiSeq high-throughput sequencing technology, we analyzed bacterial community structure and diversity characteristics to elucidate their interactions with habitat factors including plant and soil. This research aims to provide a microbiological basis for the establishment of artificial *Kengyilia thoroldiana* grasslands across diverse topographic habitats, facilitating vegetation restoration and ecological conservation. Ultimately, it seeks to offer empirical support for constructing a theoretical framework for the co-development of *Kengyilia thoroldiana* and soil bacteria.

## 2. Materials and Methods

### 2.1. Basic Characteristics of Test Area

**Figure 1:**
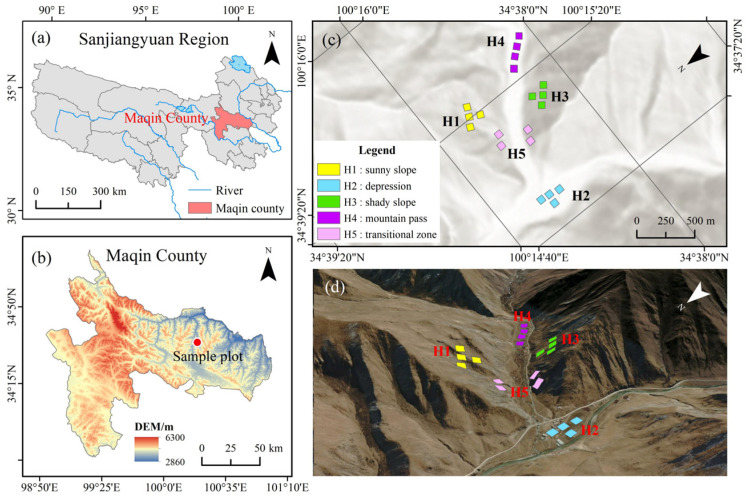
Spatial distribution characteristics of the experimental area and sampling sites. Note: (**a**) Overall scope of the Sanjiang source region, (**b**) administrative region of Maqin County, (**c**) geographic coordinate positioning of sampling sites within the study area, (**d**) three-dimensional topography of sampling sites. This figure was created based on the standard base map provided by the Standard Map Service Website of the National Administration of Surveying, Mapping and Geoinformation, China [Approval Number: GS (Beijing, China) No. 0650 (2024)].

The experimental area is located in the southeast of Qinghai Province, with a latitude and longitude range of 33°43′ to 35°16′ N and 98°24′ to 100°56′ E, and an altitude of 3800 m (Figure 1a,b). This region has typical plateau continental semi-humid climate characteristics, with an annual average temperature of about −3.5 °C and an annual average precipitation of about 500 mm [15]. The main types of ecosystems include alpine desert grasslands and alpine meadows, with soil types divided into three categories: sandy soil, sandy loam soil, and meadow soil [16]. The vegetation structure is dominated by Cyperaceae and Gramineae families, among which *Carex alatauensis*, *Kengyilia thoroldiana*, and *Poa pratensis* are dominant species, which together constitute the core components of the regional ecosystem [17].

### 2.2. Sample Plot Setting

This study employed a natural observation method, with no artificial seeding interventions conducted throughout the research process. All experimental plants were sourced from naturally occurring native populations, and their growth characteristics under natural conditions were directly reflected through systematic quadrat surveys. The specific experimental design process is as follows: In July 2023, five types of experimental zones were scientifically established within the study area based on topographic characteristics, namely sunny slope (H1), depression (H2), shady slope (H3), mountain pass (H4), and transitional zone (H5). For each topographic type, four standard transects measuring 10 m × 40 m were randomly established, resulting in a total of 20 transect units with inter-transect distances exceeding 10 m. Subsequently, within each transect, 15 measurement quadrats of 0.5 m × 0.5 m were further randomly positioned, culminating in 300 observation quadrats in total (Figure 1c,d). During the milk-ripe stage of *Kengyilia thoroldiana*, the research team conducted systematic measurements of key indicators in each sample plot, specifically including the plant community coverage, aboveground biomass, and plant height of *Kengyilia thoroldiana*, the dominant species in the community.

### 2.3. Plant Community Investigation and Soil Sample Collection

This study conducted investigations on the coverage, biomass, and plant height of *Kengyilia thoroldiana* communities during its milk-ripe stage from August to September in both 2023 and the corresponding period in 2024. Concurrently, rhizosphere soil samples of *Kengyilia thoroldiana* were collected using a “shaking-off + brushing” method: First, non-rhizosphere loose soil was gently separated from roots by light shaking; subsequently, soil particles adhering tightly to the root surface (0–4 mm) were collected using sterile brushes. For each quadrat, five original soil samples were collected using the five-point sampling method. After thoroughly mixing the original samples from the same transect, four composite soil samples were formed for each topographic type plot. From these, three composite soil samples were randomly selected from each topographic type plot for analysis, resulting in a total of 15 analytical soil samples. Each analytical soil sample was divided into two portions for preservation: one portion was stored in an ultra-low temperature freezer (MDF-86V588D, Zhongke duling, Hefei, Anhui, China) at −80 °C for soil bacterial diversity analysis, while the other portion was kept in a freezer (DW-40L92, Haier, Qingdao, China) at −20 °C for the determination of physicochemical properties, including soil organic carbon (SOC), total nitrogen (TN), total phosphorus (TP), total potassium (TK), soil water content (SWC), soil electrical conductivity (SEC), and pH.

### 2.4. Indicator Measurement

#### 2.4.1. Vegetation Characterization

The plant height of *Kengyilia thoroldiana* (measured as the vertical distance from the ground surface to the highest growth point) was determined using a measuring tape (with a precision of 1 mm). Community coverage was determined via the needle-puncture method. Plants within the quadrat were cut flush to the ground, followed by blanching in an oven (105 °C for 30 min) and drying to a constant weight (65 °C for 48 h) before weighing to obtain the aboveground biomass [18]. Data are presented as the mean values of 15 quadrats.

#### 2.4.2. Soil Characteristics

In August 2023 and the same period in 2024, during sunny morning (8:00–10:00), noon (12:00–14:00) and evening (16:00–18:00), using a TDR350 time domain reflectometer of Spectium Company in the United States (Aurora, IL), the soil conductivity and pH value were measured in the experimental plot, repeating 3 times, and monitored continuously for 3 days. Soil water content (SWC) was determined using the oven-drying method; soil organic carbon content (SOC) was measured via the potassium dichromate oxidation method; soil total nitrogen (TN) content was assayed using the Kjeldahl nitrogen determination method; soil total phosphorus (TP) content was quantified through the molybdenum-antimony colorimetric method; and soil total potassium (TK) content was determined by flame photometry [19].

#### 2.4.3. DNA Extraction, PCR Amplification, and High-Throughput Sequencing

A 0.5 g rhizosphere soil sample was collected, and genomic DNA was extracted using the Qiagen DNeasy PowerSoil Kit (Beijing, China), with all procedures conducted in strict accordance with the manufacturer’s protocol [20]. DNA integrity was assessed via 1% agarose gel electrophoresis, while DNA concentration and purity were determined using a nucleic acid analyzer (ZAG, Agilent Technologies, Santa Clara, CA, USA) to ensure subsequent library construction quality. DNA samples submitted for analysis had to meet the following quality criteria: DNA concentration ≥ 50 ng/μL with a minimum volume of 60 μL; A260/A280 ratio in the range of 1.8–2.0, indicating no significant protein or RNA contamination; and agarose gel electrophoresis demonstrating a sharp, well-defined primary band without evidence of degradation or enzymatic digestion artifacts.

The DNA samples that passed quality inspection were entrusted to Majorbio Bio-Pharm Technology Co., Ltd. (Shanghai, China) for amplification of the bacterial 16S rRNA gene V3–V4 variable region [21]. PCR amplification was performed using the classic primer pair 338F (5′-ACTCCTACGGGAGGCAGCAG-3′) and 806R (5′-GGACTACHVGGGTWTCTAAT-3′) [21]. The PCR amplification system was configured as follows: 2 μL of 10× buffer, 2 μL of 2.5 mmol/L dNTP mixture, 0.8 μL of forward primer, 0.8 μL of reverse primer, 0.2 μL of polymerase, 1 μL of template DNA, and ddH_2_O, with the total volume adjusted to 20 μL. The thermal cycling program was set as follows: initial denaturation at 95 °C for 3 min to initiate the reaction; followed by 35 cycles, each consisting of denaturation at 95 °C for 30 s, annealing at 55 °C for 30 s, and extension at 72 °C for 45 s; after the cycles, a final extension at 72 °C for 5 min was performed to fully extend the products. The final reaction system was stored at 4 °C for subsequent use. This parameter combination ensured efficient and specific amplification of the target fragment, providing high-quality templates for subsequent sequencing.

The library construction process strictly adhered to standardized operational protocols. Following verification via secondary agarose gel electrophoresis, target bands were precisely recovered using the Qiagen Gel Extraction Kit (Qiagen, Düsseldorf, Germany). Subsequently, library construction was completed using the NEBNext^®^ UltimaTM II DNA Library Prep Kit (New England Biolabs, Ipswich, MA, USA), with quantitative assessment performed through Qubit fluorometry and Q-PCR. Libraries that passed quality control were subjected to paired-end sequencing on the NovaSeq 6000 high-throughput sequencing platform (Illumina, San Diego, CA, USA). The raw sequencing data underwent rigorous quality control procedures, including the removal of chimeric sequences, prior to proceeding with downstream bioinformatics analysis.

During the bioinformatics analysis phase, high-quality operational taxonomic unit (OTU) representative sequences and feature abundance tables were generated based on a 97% sequence similarity threshold. Subsequently, OTU sequences were aligned and annotated against the authoritative Silva138.1 database using the sklearn classification module in QIIME 2 (2024.10 version) to determine microbial species affiliations. Throughout the analysis, OTU sequences derived from chloroplasts and mitochondria were systematically removed, and data rarefaction normalization was implemented to ensure uniform sequencing depth across samples, thereby completing the microbial community structure analysis. The sequencing data processing and species annotation in this phase were exclusively performed by the professional team at Majorbio Bio-Pharm Technology Co., Ltd. (Shanghai, China), ensuring end-to-end quality control from experimental operations to bioinformatics analysis.

### 2.5. Data Processing and Statistical Analysis

Preliminary data organization, calculations, and standard deviation processing for vegetation and soil data were conducted using Microsoft Excel 2019 software (Microsoft, Washington, DC, USA). Normality and homogeneity of variance tests for the vegetation and soil data were performed using SPSS 27.0 software (IBM, New York, NY, USA). For data meeting the assumptions of normal distribution and homogeneity of variance, one-way analysis of variance (one-way ANOVA) and least significant difference (LSD) tests were conducted.

Additionally, systematic visualization analysis of soil bacterial communities was performed via the Majorbio Cloud Platform (https://magic.novogene.com, accessed on 20 May 2025), encompassing Venn analysis, phylum- and genus-level abundance analysis, linear discriminant analysis effect size (LEfSe) analysis, α/β diversity analysis, hierarchical clustering analysis, and FAPROTAX functional prediction. Dilution curves were generated using QIIME 2 software to validate the rationality of sequencing depth, while phylum- and genus-level species relative abundance charts were constructed to visually present the distribution characteristics of dominant taxa. Community diversity was quantified using α-diversity indices (including OTU richness, Shannon index, Simpson index, Pielou evenness index, Chao1 index, and ACE index). β-diversity analysis was performed using Bray–Curtis distance-based principal coordinate analysis (PCoA) plots to reveal inter-sample community structure differences, with hierarchical clustering dendrograms employed to verify grouping consistency. To further investigate differences in community structure among grouped samples, the linear discriminant analysis effect Size (LEfSe) statistical method was selected to conduct significance testing for species composition and community structure disparities across sample groups. Utilizing the FAPROTAX functional prediction software (1.2.12), OTU annotation results were correlated with functional databases to predict the ecological functional potential of microbial communities. Single-factor molecular network diagrams were constructed using NetworkX (version 1.11) to quantify interspecific association strengths. Pearson correlation coefficients were employed to generate heatmaps on the R 3.5.2 platform, illustrating relationships between plant-soil traits and bacterial community diversity, phylum-level bacterial community abundance, and genus-level bacterial community abundance. Redundancy analysis (RDA) was conducted using Canoco 5.0 software (Microcomputer Power, New York, NY, USA) to quantify the explanatory power of plant-soil traits on bacterial community diversity, phylum-level abundance, and genus-level abundance, thereby clarifying the driving effects of environmental factors on bacterial community structure.

## 3. Results and Analysis

### 3.1. 5 Characteristics of Rhizosphere Soil and Vegetation Community of Kengyilia thoroldiana in Five Topographic Environments

As shown in Table 1, there are significant differences in plant community coverage, plant community biomass, and plant height of *Kengyilia thoroldiana* under different topographic conditions. The coverage of plant communities ranged from 35.11% to 70.81%, and the specific order was H2 > H5 > H4 > H3 > H1. The coverage of habitat H2 was the highest, reaching 70.81, followed by that of habitat H5, reaching 70.25. There was no significant difference between them, but both were significantly higher than that of habitat H1 (*p* < 0.05). The plant height in H2 reached the maximum, which was 34.87 cm, while that in H1 reached the minimum, which was only 18.63 cm, significant difference (*p* < 0.05). The biomass of habitat H2 was the highest, at 652.49 g·m^−2^, which was 88.47% higher than that of habitat H1, a significant difference (*p* < 0.05).

At the same time, the study identified significant differences in the soil physical and chemical properties of the five types of topographic habitats (Table 1). The moisture content of *Kengyilia thoroldiana* rhizosphere soil in the five topographic habitats ranged from 23.13% to 40.38%, and the descending order of moisture content was H2 > H5 > H4 > H3 > H1. The SEC in habitat H2 reached the lowest value of 599.41 μs·cm^−1^, while that in habitat H1 reached the maximum value of 1098.36 μs·cm^−1^. Compared with habitat H1, the SEC of H2 decreased by 45.43% (*p* < 0.05). The pH value of the soil ranged from 7.97 to 8.66, and that of habitat H2 was the lowest, which was significantly lower than the other habitats except H5. Among the five topographic habitats, the soil organic carbon content ranged from 5.60 to 15.30 g·kg^−1^, and the organic carbon content in habitat H2 was the highest, at 15.30 g·kg^−1^, an increase of 173.21% compared with that in habitat H1 and a significant difference (*p* < 0.05). The total nitrogen content was the highest in habitat H2, at 1.55 g·kg^−1^, which was 101.30% higher than that in habitat H1. The TP content in habitat H2 was 0.58 g·kg^−1^, which was significantly higher than that in habitat H1, by 81.25% (*p* < 0.05). The TK content of oat rhizosphere soil in the five topographic habitats ranged from 19.08 g·kg^−1^ to 25.28 g·kg^−1^, with the highest value of 25.28 g·kg^−1^ in habitat H2 and a value of 22.05 g·kg^−1^ in habitat H5.

### 3.2. 5 Sequencing Quality, Venn Analysis, and Species Composition of Bacteria in Kengyilia thoroldiana Rhizosphere Soil Samples

#### 3.2.1. Sequencing Results and Changes in Soil Bacterial OTU Quantity

**Figure 2:**
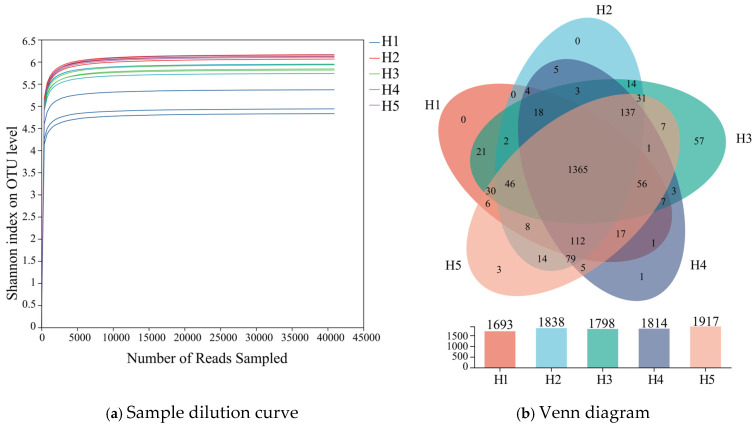
Dilution curve and Venn diagram of *Kengyilia thoroldiana* rhizosphere soil samples. Note: (**a**) Dilution curves, (**b**) Venn diagrams. The abscissa represents the amount of sequencing data randomly selected; the ordinate represents the observed Shannon index.

As illustrated in Figure 2a, with the increase in sample size, the rarefaction curves corresponding to the five types of terrain habitats gradually flattened out when sequencing reads reached approximately 12,000–15,000. This indicated that the current sequencing data volume (13,500 reads per sample) had approached saturation, with this depth covering over 95% of prevalent bacterial taxa. The sequencing depth setting was therefore deemed appropriate. Further expansion of data volume beyond 20,000 reads would only enable identification of a minimal number of low-abundance species. Consequently, the sequencing results from existing soil samples could reliably reflect the authentic status of bacterial communities across the five terrain habitats.

Venn analyses of bacterial operational taxonomic units (OTUs) were carried out on inter-root soil samples from the five types of topographic habitats of *Kengyilia thoroldiana*, and the results are shown in Figure 2b. A total of 2052 bacterial OTUs were identified in the five types of habitats, with 1693, 1838, 1798, 1814, and 1917 OTUs in habitats H1, H2, H3, H4, and H5, respectively. The number of OTUs common to the five habitat types amounted to 1365, or 66.49% of the total number of OTUs. In terms of OTU habitat specificity, no unique OTU was detected in habitats H1 and H2, while 57, 1, and 3 unique OTUs were detected in habitats H3, H4, and H5, accounting for 2.78%, 0.05%, and 0.15%, respectively.

#### 3.2.2. Analysis of Bacterial Community Composition and Relative Abundance

**Figure 3:**
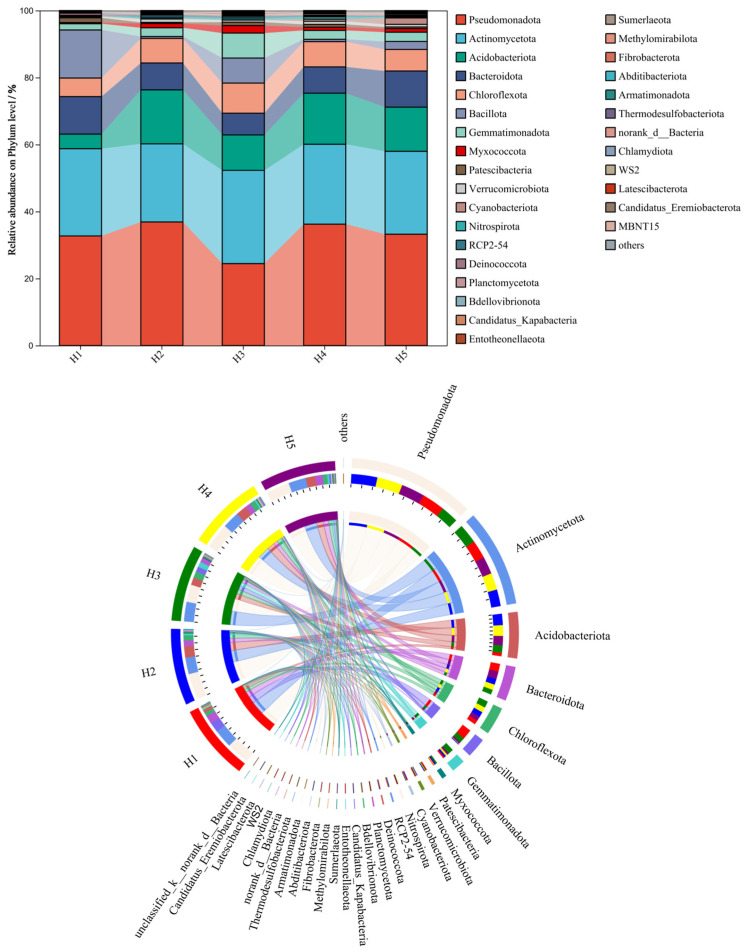
Relative abundance of bacterial phyla at the phylum level across five topographic habitats. Note: The data on the proportion of bacterial abundance are expressed as average values (*n* = 3).

Figure 3 illustrates the relative abundance distribution of the top 30 bacterial phyla in rhizosphere soil samples of *Kengyilia thoroldiana* across five habitat types. Among the soil bacteria at the phylum level, Pseudomonadota (24.45~35.89%), Actinobacteriota (23.32~27.83%), Acidobacteriota (4.36~16.13%), Bacteroidota (6.49~11.20%), and Chloroflexi (5.54~8.99%) were relatively more abundant. The relative abundance ranking of soil bacterial phyla was approximately the same for the five types of topographic habitats, with Pseudomonadota, Actinobacteriota, and Acidobacteriota being the dominant communities in each sample. Habitats H2 and H5 had an increased abundance of the Pseudomonadota phylum and Acidobacteriota phylum and decreased abundance of the Acidobacteriota phylum in soil bacteria compared to habitats H1 and H3. At the same time, compared to habitat H1, all other habitat types had an increased abundance of the Chloroflexi phylum and decreased abundance of the Bacteroidota phylum of soil bacteria.

**Figure 4:**
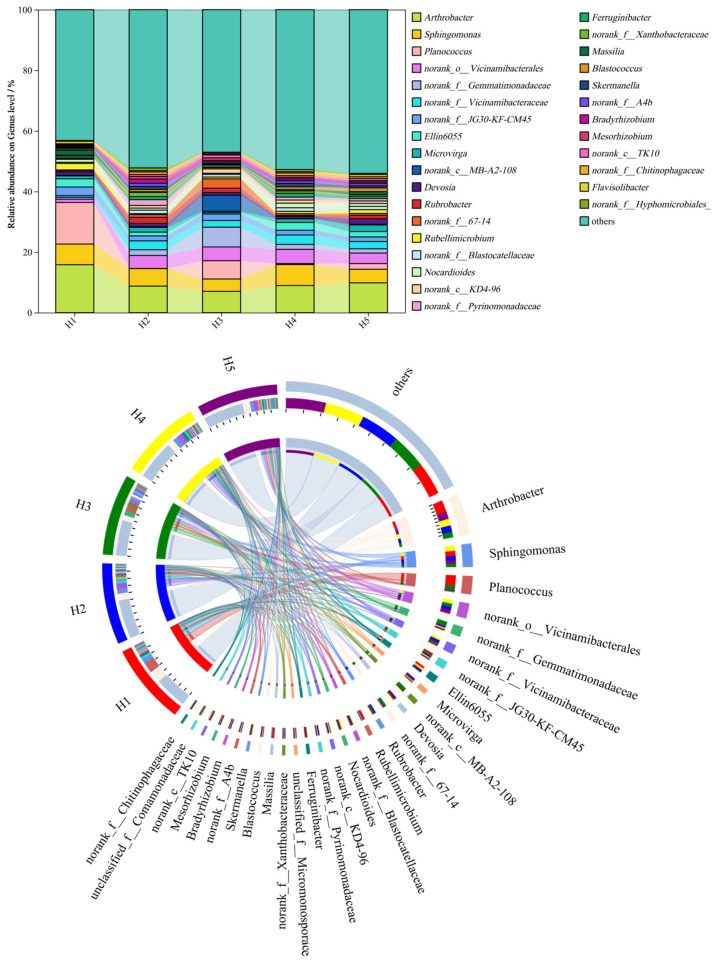
Relative abundance of bacterial genera at the genus level across five topographic habitats. Note: The data on the proportion of bacterial abundance are expressed as average values (*n* = 3).

Figure 4 shows the relative abundance distribution of the top 30 bacterial genera in rhizosphere soil samples of *Kengyilia thoroldiana* across five habitat types. Among the soil bacteria at the genus level, *Arthrobacter* (7.04~15.84%), *Sphingomonas* (4.09~6.93%), *Planococcus* (0.00~13.72%), norank_o__*Vicinamibacterales* (1.09~4.72%), and norank_f__*Gemmatimonadaceae* (0.60~6.53%) were the relatively abundant bacterial genera in the five types of topographic habitats. There were obvious differences in the relative abundance order of different bacterial genera in the five types of topographic habitats. Compared with habitat H1, the other four habitats had an increased abundance of norank_o__*Vicinamibacterales* and norank_f__*Gemmatimonadaceae* and decreased abundance of *Arthrobacter* and *Planococcus*.

### 3.3. Bacterial Community LEfSe Analysis

**Figure 5:**
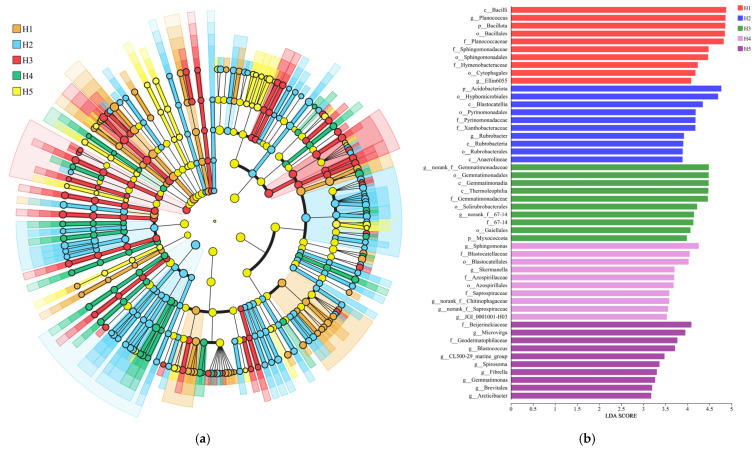
LEfSe analysis of *Kengyilia thoroldiana* rhizosphere soil samples. Note: (**a**) LEfSe analysis (LDA > 3.0); (**b**) indicator species LDA values (LDA > 3.0).

As shown in Figure 5a, LEfSe analyses allowed the identification of indicator species (LDA values above 3.0) that contributed most significantly to differences in bacterial communities across the five topographic habitat categories, and such analyses provided a clear picture of the bacterial categories that significantly influenced changes in community structure. Based on the information in Figure 5a,b, 50 biomarkers (10 each in habitats H1, H2, H3, H4, and H5) were identified by the bacterial community in the five types of topographic habitats. At the taxonomic level, the highest-scoring biomarkers in habitats H1, H2, H3, H4, and H5 were c__Bacilli (4.879), p__Acidobacteriota (4.769), g__norank__f__Gemmatimonadaceae (4.485), g__Sphingomonas (4.257), and f__Beijerinckiaceae (4.089) (Figure 5b).

### 3.4. Differences in Diversity of Soil Bacterial Communities in Five Topographic Types of Kengyilia thoroldiana Habitats

#### 3.4.1. Variations in α Diversity of Bacteria in *Kengyilia thoroldiana* Rhizosphere Soil Samples

**Figure 6:**
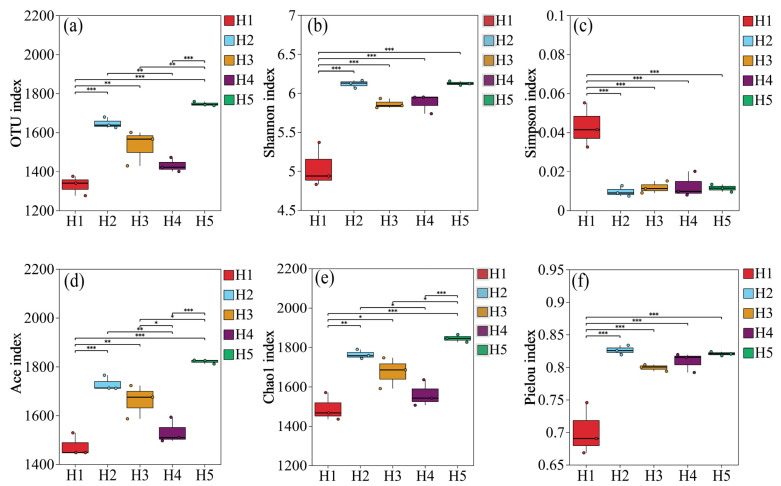
Diversity of bacterial communities in *Kengyilia thoroldiana* rhizosphere soil samples. Note: (**a**) number of OTUs, (**b**) Shannon index, (**c**) Simpson index, (**d**) Ace index, (**e**) Chao1 index, (**f**) Pielou index. * represents *p* < 0.05, ** represents *p* < 0.01, *** represents *p* < 0.001.

As can be seen from Figure 6, there were significant differences in the α diversity index of soil bacterial communities in the rhizosphere of *Kengyilia thoroldiana* in five topographic habitats. Specifically, the number of soil bacterial OTUs and Shannon index in habitat H5 reached the highest values, which were 1746.33 and 6.13, respectively, significantly higher than those in habitat H1 (*p* < 0.05), with increases of 31.27% and 21.63%, respectively. In terms of the Simpson index, the five habitats were ranked as habitat H1 > H4 > H3 > H5 > H2, where the differences among habitats H2, H3, H4 and H5 were not significant (*p* > 0.05); the Simpson indices of the above four habitats were all lower than that of habitat H1 (*p* < 0.05). In addition, the Ace index and Chao1 index of habitat H5 were the highest, at 1820.85 and 1844.80, respectively, which were 23.48% and 23.76% higher than those of habitat H1, with significant differences (*p* < 0.05). In terms of the Pielou index of the five topographic habitats, that of habitat H2 was the highest, at 0.83, which was 18.57% higher than that of habitat H1 (*p* < 0.05). To sum up, compared with habitat H1, habitats H2 and H5 had significantly increased numbers of OTUs of soil bacteria in *Kengyilia thoroldiana* rhizosphere and showed improved values for other diversity indexes except for the Simpson index.

#### 3.4.2. Analysis of β Diversity (PCoA) of Bacterial Community

**Figure 7:**
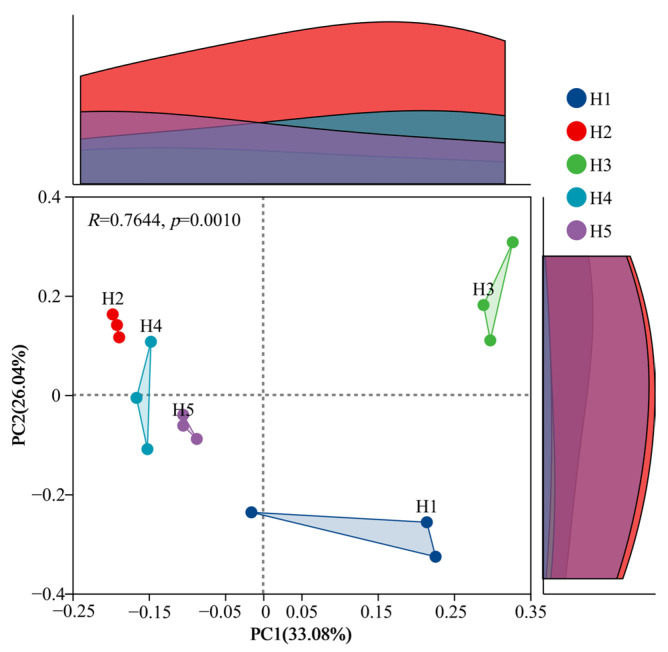
PCoA analysis of bacteria in *Kengyilia thoroldiana* rhizosphere soil samples.

Based on the Bray–Curtis distance, the bacterial composition of *Kengyilia thoroldiana* rhizosphere soil in the five types of topographic habitats was analyzed by PCoA, and the results are shown in Figure 7. Soil bacteria were most highly aggregated in habitats H2 and H5, with the least within-group variation. Habitat H1 had the lowest aggregation and the largest intra-group variation. The interpretation rate of the PC1 axis was 33.08%, that of the PC2 axis was 26.04%, and the cumulative interpretation rate was 59.12%, with significant differences between groups (*p* = 0.001). The PC1 axis clearly distinguished the bacterial communities of H1, H2, H3, H4 and H5 habitats, suggesting that PC1 was the main factor contributing to the differences in the inter-root bacterial communities of *Kengyilia thoroldiana* in the five types of topographic habitats.

#### 3.4.3. Clustering Characteristics of Soil Bacterial Communities

**Figure 8:**
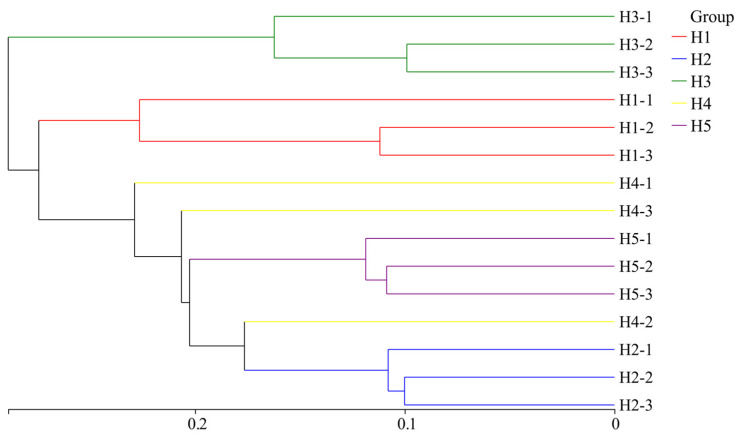
Cluster analysis of soil bacterial communities in the *Kengyilia thoroldiana* rhizosphere.

Based on the hierarchical clustering distance, 15 samples of *Kengyilia thoroldiana* soil were clustered. As shown in the hierarchical cluster analysis of samples (Figure 8), the bacteria in the five types of topographic habitats could be divided into two groups: habitat H3 formed one group independently (Group 1), and the other four topographic habitats belonged to the other group (Group 2). Further analysis showed that when the distance parameter was set to 0.28, Group 2 could be subdivided into two subgroups, in which habitat H1 alone constituted a subgroup, while habitats H2, H4, and H5 together constituted the other subgroup. In addition, the bacterial communities of three *Kengyilia thoroldiana* rhizosphere soil samples collected from habitats H1, H2, H3, and H5 each clustered into a single clade, which indicates that the soil bacterial community structure in these habitats had high similarity. In contrast, that of habitat H4 was distributed in different branches, which shows that the composition of its soil bacterial community was different.

### 3.5. Single-Factor Molecular Network Analysis of Bacteria in Kengyilia thoroldiana Rhizosphere Soil Samples

**Figure 9:**
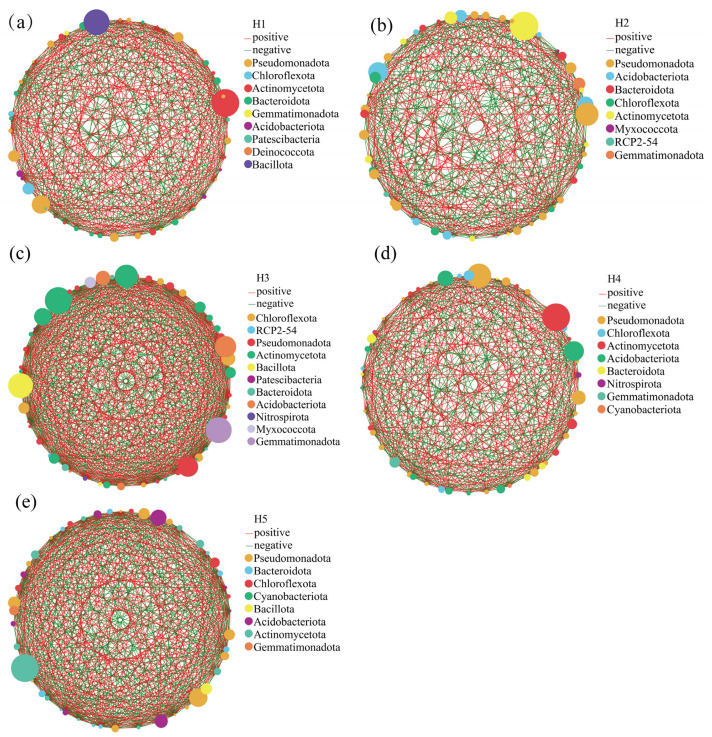
Single-factor molecular network analysis of rhizosphere soil bacteria from five habitats of *Kengyilia thoroldiana*. Note: Node color and size indicate species type and importance; line color indicates positive or negative correlation, with red positive and green negative; and the number of lines indicates whether the species are closely related. (**a**) H1, (**b**) H2, (**c**) H3, (**d**) H4, (**e**) H5.

In order to investigate the co-occurrence characteristics of bacterial genus levels and inter-community interactions in the inter-root soils of *Kengyilia thoroldiana* in five topographic environments, a one-factor correlation network of soil bacteria based on the genus level was constructed in this study (Figure 9 and Supplementary Table S1). The results showed significant differences in network topology among the five topographic habitats. Specifically, the number of edges in the habitat H3 network was the largest (618), followed by habitat H5 (548), while the number of edges in habitat H1 was 426, which was 31.08% lower than that in habitat H3. In contrast, there was less variation in the number of network nodes between habitats. From the perspective of correlation, the positive correlation of the habitat H1 network was the highest, at 56.57%, while the positive correlations of habitats H2 and H5 were lower than 50.00%, at 48.47% and 49.45% respectively. In the bacterial one-factor correlation network, the key nodes differed among the five habitats: habitat H1 was centered on Pseudomonadota, Chloroflexota, and Actinomycetota; H2 was dominated by Pseudomonadota, Acidobacteriota, and Bacteroidota; the key nodes of H3 were Chloroflexota, RCP2-54, and Pseudomonadota; H4 had Pseudomonadota, Chloroflexota, and Actinomycetota as important nodes; and the core phyla of H5 were Pseudomonadota, Bacteroidota, and Chloroflexota.

### 3.6. Prediction of Soil Bacterial Community Function in Kengyilia thoroldiana Rhizosphere Soil Samples from Five Types of Topographic Habitats

**Figure 10:**
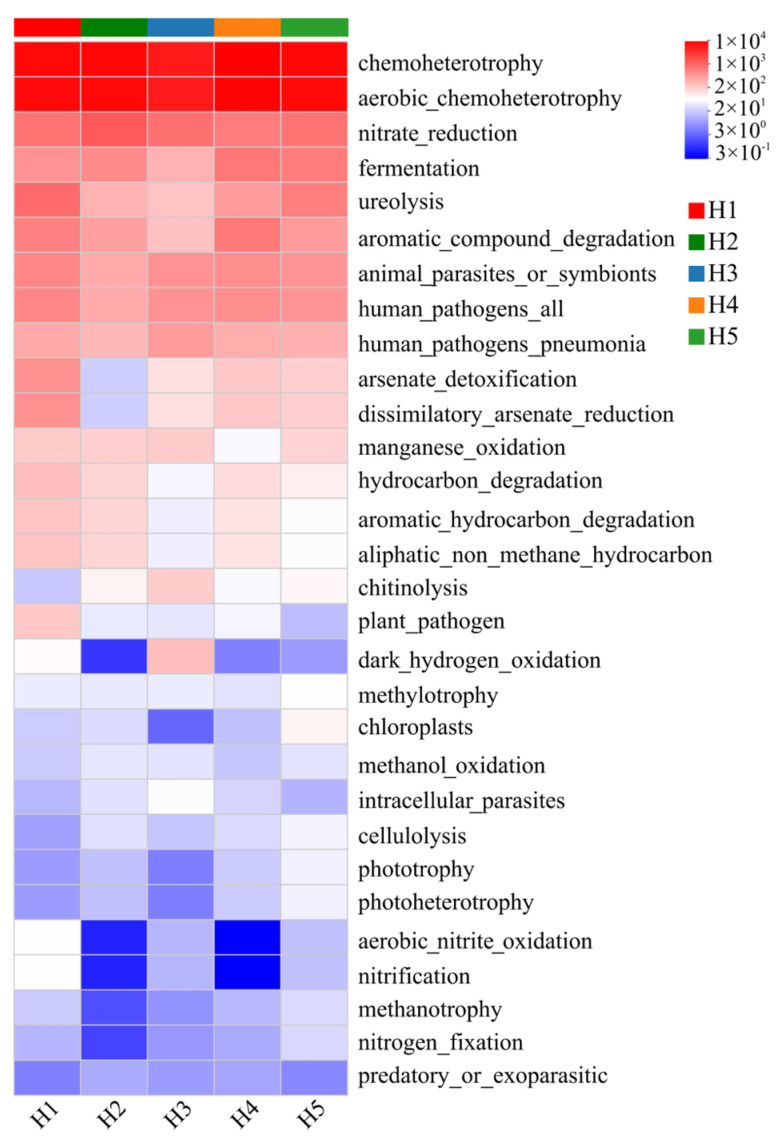
Heatmap of FAPROTAX function prediction of soil bacterial community in *Kengyilia thoroldiana* rhizosphere soil samples. Note: The data represent mean values, n = 3. The abscissa denotes sample names, while the ordinate indicates functional categories. Color gradients in the heatmap represent variations in functional abundances across samples, with the numerical scale corresponding to color intensities shown on the right side of the figure.

Based on the FAPROTAX tool, a functional annotation of bacterial communities in *Kengyilia thoroldiana* rhizosphere soil was carried out, and 30 sub-functions were identified. As shown in Figure 10, chemoheterotrophy and aerobic_chemoheterotrophy were dominant in the five types of topographic habitats, and especially in H2, H4 and H5, the functional intensity was significantly higher than in other topographic habitats (higher than 9300). Specific to the differentiation characteristics of each habitat, the light energy utilization function (phototrophy, photoheterotrophy) and predatory function (predatory _ or _ exotic) of habitat H1 were the weakest (<10). There were obvious shortcomings in the functions of dark_hydrogen_oxidation, aerobic_nitrite_oxidation, nitrification, methane metabolism, and nitrogen_fixation in habitat H2. Chloroplast-related functions (chloroplasts), light energy utilization (phototrophy, photoheterotrophy), nitrogen_fixation, methanotrophy, and predatory_or _exoparasitic all showed weakening characteristics; habitat H4 was deficient in dark_hydrogen_oxidation, aerobic_nitrite_oxidation, nitrification, and predatory_or_exoparasitic functions; H5 mainly had functional defects in dark_hydrogen_oxidation and predatory_or_exotic.

### 3.7. Correlations Between Rhizosphere Soil Bacterial Community and Habitat Factors of Kengyilia thoroldiana

#### 3.7.1. Mantel Test Analysis of Rhizosphere Soil Bacterial Community and Habitat Factors of *Kengyilia thoroldiana*

**Figure 11:**
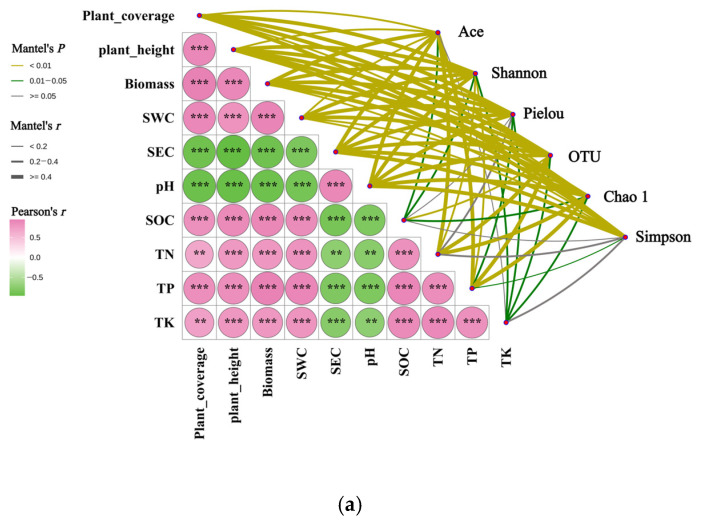

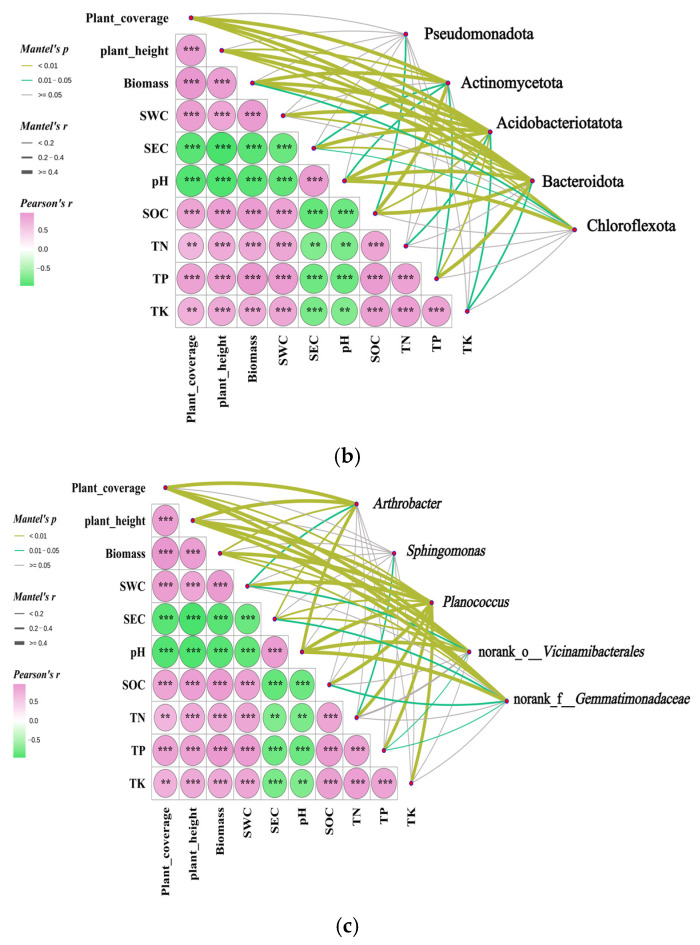
Mantel test analysis of the correlation between rhizosphere soil bacterial community and habitat factors of *Kengyilia thoroldiana.* Note: ** denotes *p* < 0.01, and *** denotes *p* < 0.001. (**a**) α diversity, (**b**) bacterial phylum level, and (**c**) bacterial genus level.

The Mantel test revealed extensive and statistically significant associations between three plant community indices and seven soil physicochemical properties (Figure 11). SEC exhibited an extremely statistically significant positive correlation with pH (*p* < 0.001). Both SEC and pH demonstrated highly statistically significant (*p* < 0.01) or extremely statistically significant negative (*p* < 0.001) correlations with the other three plant indices and five soil properties. The remaining eight pairs of vegetation-soil indices displayed highly statistically significant (*p* < 0.01) or extremely statistically significant positive (*p* < 0.001) correlations.

In addition, the above 10 plant and soil indicators showed significant correlations with soil bacterial community structure (including alpha diversity, phylum-level abundance, and genus-level abundance), as illustrated in Figure 11. Specifically, at the alpha diversity level (Figure 11a), the Ace index showed correlations with all vegetation-soil factors except TK (*p* < 0.05). The Pielou index also exhibited correlations with all vegetation-soil factors except SOC and TN (*p* < 0.05). The Shannon index, OTU number, and Chao1 index demonstrated correlations with all 10 vegetation-soil factors (*p* < 0.05). The Simpson index displayed significant (*p* < 0.05) or highly significant (*p* < 0.01) correlations with three vegetation characteristics and four soil factors, excluding SOC, TN, and TK.

At the phylum level (Figure 11b), Pseudomonadota abundance was significantly correlated with SOC (*p* < 0.05). Actinomycetota abundance showed significant (*p* < 0.05) or highly significant (*p* < 0.01) correlations with seven vegetation-soil indicators, excluding SWC, TN, and TK. Acidobacteriota showed significant (*p* < 0.05) or highly significant (*p* < 0.01) correlations with all 10 vegetation-soil indicators. The abundance of Bacteroidota exhibited significant (*p* < 0.05) or highly significant (*p* < 0.01) correlations with nine vegetation-soil indicators, excluding TN. The abundance of Chloroflexota demonstrated significant correlations (*p* < 0.05) with biomass and SEC, and highly significant correlations (*p* < 0.01) with plant coverage, plant height, and pH.

At the genus level (Figure 11c), the abundance of *Arthrobacter* showed a significant correlation with SWC (*p* < 0.05) and a highly significant correlation with plant coverage, plant height, biomass, SEC, and pH (*p* < 0.01). The abundance of *Sphingomonas* was significantly correlated with TN (*p* < 0.05). The abundance of *Planococcus* exhibited a highly significant correlation with all 10 vegetation-soil indicators (*p* < 0.01). The abundance of Norank-o-*Vicinamibacterales* showed significant (*p* < 0.05) or highly significant (*p* < 0.01) correlations with seven vegetation-soil indicators, excluding SOC, TN, and TK. The abundance of Norank-f-*Gemmatimonadaceae* was significantly correlated with SEC, SOC, and TP (*p* < 0.05), and highly significantly correlated with plant coverage, plant height, and pH (*p* < 0.01).

#### 3.7.2. Redundance Analysis of the Relationship Between Rhizosphere Soil Bacterial Community and Habitat Factors of *Kengyilia thoroldiana*

**Figure 12:**
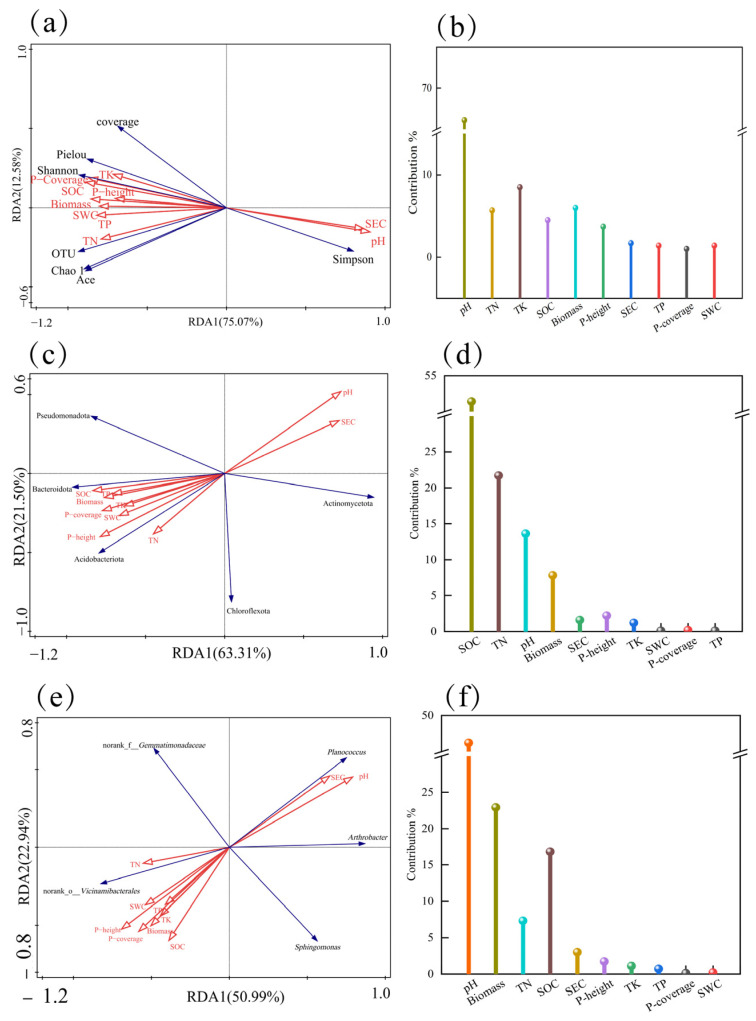
RDA analysis of soil physical and chemical properties, plant community characteristics, and soil bacterial community diversity in the *Kengyilia thoroldiana* rhizosphere. Note: Red arrows indicate plant community characteristics and soil factors, and blue arrows indicate the fungal community structure. (**a**,**b**) α diversity; (**c**,**d**) bacterial phylum level; (**e**,**f**) bacterial genus level.

The explanation rates of bacterial community α-diversity by vegetation-soil characteristics on Axis I and Axis II were 75.07% and 12.58%, respectively, with a cumulative rate of 87.65% (Figure 12a,b). Among them, soil pH had the highest contribution rate (66.1%) and was significantly influential (*p* = 0.002), serving as the most critical factor affecting the α-diversity of bacterial communities in the rhizosphere soil of *Kengyilia thoroldiana*. Further analysis revealed that SEC and pH were positively correlated with the Simpson index, while negatively correlated with the Pielou index, Shannon index, OTU number, Chao1 index, and Ace index. The remaining eight vegetation-soil factors showed positive correlations with all other soil bacterial diversity indices except for the Simpson index.

The explanation rates of soil bacterial community phylum-level abundance by vegetation-soil characteristics on the first and second principal axes were 63.31% and 21.50%, respectively, with a cumulative rate of 84.81% (Figure 12c,d). SOC was identified as the primary driving factor (contributing 51.4%, *p* = 0.002), and its spatial heterogeneity significantly influenced the composition of bacterial phyla. TN ranked second (contributing 21.7%), indicating that soil nutrients have a significant impact on the phylum-level composition of bacterial communities. The abundance of Actinomycetota was positively correlated with pH and electrical conductivity, while negatively correlated with the other eight vegetation-soil indicators.

The explanation rates of soil bacterial community genus-level abundance by vegetation-soil characteristics on the first and second principal axes were 50.99% and 22.94%, respectively, with a cumulative rate of 73.93% (Figure 12e,f). pH was identified as the primary driving factor (contributing 46.2%, *p* = 0.006), and its spatial heterogeneity significantly influenced the composition of bacterial genera, indicating that soil acidity-alkalinity has a notable impact on the genus-level community. The abundances of *Arthrobacter* and *Planococcus* were positively correlated with pH and electrical conductivity, while negatively correlated with the other eight vegetation-soil indicators.

## 4. Discussion

### 4.1. Changes in Soil Physical and Chemical Properties and Vegetation Community Characteristics in Kengyilia thoroldiana Rhizosphere Samples from Five Types of Topographic Habitats

Soil physical and chemical properties, such as pH value, electrical conductivity, and water content, have a significant impact on soil nutrient status, and soil nutrient content, including organic carbon, nitrogen, phosphorus, and potassium, is an important indicator for the measurement of soil quality [22]. Topographic differences significantly affect the physical and chemical properties of *Kengyilia thoroldiana* rhizosphere soil and vegetation growth: the contents of nutrients such as organic carbon, total nitrogen, total phosphorus, and total potassium in *Kengyilia thoroldiana* rhizosphere soil in the depression and transitional zone habitats are high, the biomass of the plant community is large, and the coverage is high. The soil nutrient content of the *Kengyilia thoroldiana* rhizosphere on shady and sunny slopes is low, the surface is bare, and the community vegetation is scarce. These findings are consistent with the research results of Liu et al. [23] on the vegetation characteristics of sparse grassland in the Hunshandake area; that is, in three types of topographic habitats, the soil water content, organic matter content, and total nitrogen content are the highest in the flat habitat, followed by the shady slope habitat, and the lowest in the sunny slope habitat. The reasons for these differences lie in the fact that the depressions and transitional zones feature gentle slopes and favorable microclimates, which are more conducive to plant growth. Among them, the *Kengyilia thoroldiana* community exhibits relatively high coverage, effectively conserving soil and water, reducing soil nutrient loss, and thereby enhancing soil organic matter and moisture content [23,24]. Meanwhile, after the decomposition of plant litter such as branches and leaves in the community, abundant nutrients can be transported to the soil, further improving the fertility and water-holding capacity of the rhizosphere soil of *Kengyilia thoroldiana* [23,24]. Li et al. [25] demonstrated that significant differences exist in vegetation and soil characteristics across different topographic habitats—vegetation height, biomass, as well as soil moisture content and nutrient contents such as nitrogen, phosphorus, and potassium in the floodplain habitat are markedly higher than those in sunny and shady slope habitats. In summary, vegetation restoration and soil nutrient enrichment can form a virtuous cycle: vegetation restoration promotes soil nutrient accumulation, while soil nutrient enrichment, in turn, nourishes vegetation growth, ultimately achieving positive ecological succession [23,24,25].

### 4.2. Composition and Structural Characteristics of Bacterial Community in Kengyilia thoroldiana Rhizosphere Soil in Five Topographic Habitats

Among the five types of topographic habitats in the Yellow River source basin, significant differences exist in the water content, pH, electrical conductivity, and nutrient contents (such as carbon, nitrogen, phosphorus, and potassium) in the rhizosphere soil of *Kengyilia thoroldiana*. This habitat heterogeneity directly shapes differentiated soil bacterial community structures. Specifically, the number of bacterial operational taxonomic units (OTUs) in the rhizosphere soil of *Kengyilia thoroldiana* in depression and transitional zone habitats (1838 and 1917, respectively) is significantly higher than that in shady and sunny slope habitats. This variation may arise from two underlying mechanisms: Firstly, the depressions and transitional zone habitats feature gentle terrain and are situated between shady and sunny slopes. Wind and runoff can transport litter, soil particles, and adhering microorganisms from the shady and sunny slopes to these areas, directly augmenting the input of rhizosphere microorganisms [25,26]. Secondly, the soil in the depressions and transitional zone habitats exhibits near-neutral pH, high water content, and abundant nutrients. These favorable conditions of acidity–alkalinity, moisture, and nutrient availability can mitigate interbacterial competition intensity, providing a superior environment for microbial survival and reproduction, thereby facilitating their persistence and colonization [27,28].

Among the five types of topographic habitats in the Yellow River Source Basin Unit, Pseudomonadota, Actinobacteriota, and Acidobacteriota were the three types of bacterial phyla with high relative abundance in the *Kengyilia thoroldiana* rhizosphere, suggesting that they have important functions and roles [29]. Numerous studies have shown that there are significant differences in soil nutrient content in different topographic habitats, which in turn lead to differences in the composition of soil bacterial communities [30,31,32]. Wang et al. [33] and Zou et al. [34] found that the relative abundance of the Acidobacteriota phylum and Chloroflexi phylum was higher in topographic habitats with fertile soil and healthy vegetation communities. The reason for this may be related to the nutritional properties of these two groups of bacteria: Acidobacteriota and Chloroflexi are nutrient-rich bacterial phyla that promote humus decomposition, and their relative abundance is usually positively correlated with soil nutrient content [33,34]. In this study, the relative abundance of the phyla Acidobacteriota and Chloroflexi in the rhizosphere soil of *Kengyilia thoroldiana* in the depression and transitional zone habitats was significantly higher than that in the sunny slope habitat, which was consistent with the above research results.

The results of this study showed that the alpha diversity of bacteria in *Kengyilia thoroldiana* rhizosphere soil was significantly different in five topographic habitats in the Yellow River source basin. Specifically, compared with the sunny slope habitat, the Shannon index, Ace index, Chao 1 index, and Pielou index of bacteria in the depression habitat were significantly improved. This result is consistent with the reports of Li et al. [25] and Jiang et al. [35], further confirming the significant regulatory effect of topographic habitat on soil bacterial alpha diversity. This effect may come from differences in soil physical and chemical properties (such as water content and soil nutrients), and may also be related to the structure and function of plant communities [25,35]. According to PCoA analysis, the composition and structure of soil bacteria in different topographic habitats had different characteristics. Among them, the composition of soil bacteria in the H2 and H5 topographic habitats was relatively aggregated, while the aggregation in habitats H1 and H3 was relatively low. This phenomenon may stem from differences in soil physicochemical properties in different topographic habitats, which affect *Kengyilia thoroldiana* root development and plant growth processes, ultimately leading to significant changes in soil bacterial community structure [30,31,36]. The results of cluster analysis revealed that the rhizosphere soil bacteria in the tested *Kengyilia thoroldiana* soil samples were categorized into two groups: habitat H3 formed one group (Group 1), while the remaining habitats constituted the other group (Group 2). Furthermore, Group 2 could be subdivided into two subgroups, with habitat H1 representing one subgroup and the other habitats comprising the second subgroup. This grouping pattern may be associated with differences in soil salinity-alkalinity characteristics [37], as elevated soil pH and electrical conductivity can impose salinity-alkalinity stress on soil bacteria, thereby influencing their colonization and survival [38,39].

The interactions among soil bacteria represent a critical dimension for evaluating their community structure and function, with network analysis offering an effective means to uncover the association patterns and stability of different bacterial taxa within the community [40,41]. This study systematically elucidated the symbiotic characteristics of rhizosphere soil bacteria associated with *Kengyilia thoroldiana* across five types of topographic habitats in the Yellow River source basin through single-factor correlation network analysis. The results revealed that the bacterial network in shady slope habitats exhibited higher complexity, with predominantly positive correlations, indicating that most coexisting bacterial taxa in this environment demonstrated mutualistic or synergistic cooperative patterns. Such a community structure facilitates efficient nutrient acquisition by plant roots [25,42,43]. Further analysis revealed that Proteobacteria and Actinobacteriota occupied central positions across the five types of topographic habitats. By stabilizing community structure, improving soil conditions, and enhancing plant productivity, these two phyla emerged as key taxonomic groups essential for maintaining the ecological functions of the rhizosphere soil of *Kengyilia thoroldiana* in the Yellow River source basin [44].

The study by Li et al. [45] revealed that changes in the structure of bacterial communities in the rhizosphere soil of plants may trigger changes in their functions. In this study, FAPROTAX was used to predict the function of soil bacteria in the rhizosphere of *Kengyilia thoroldiana*, and it was found that the function of soil bacteria in five types of topographic habitats was obviously different, which was consistent with the research results. Meanwhile, it was found that chemoheterotrophy and aerobic_chemoheterotrophy functions were the strongest in all five types of topographic habitats. This may be due to the fact that soil bacteria are closely related to carbon cycling and organic matter decomposition, and surface apoplastic litter can increase soil carbon input, providing substrates for chemoheterotrophy and aerobic_chemoheterotrophy bacteria and promoting their growth and reproduction [46,47]. Compared to the other four topographic habitats, sunny slope habitats were weaker in light-energy-utilizing class functions (phototrophy, photoheterotrophy) and predatory functions (predatory_or_exoparasitic). This may be due to the poor soil and low organic carbon content in sunny slope habitats, which makes it difficult for light-using bacteria to obtain sufficient carbon sources for photosynthesis, thus limiting their phototrophic functioning to the detriment of their growth and reproduction [48,49].

### 4.3. Coupling Relationships Between Bacterial Community Structure in Kengyilia thoroldiana Rhizosphere Soil and Plant and Soil Factors

Mantel test analysis showed a significant positive correlation between the coverage, biomass, and plant height of *Kengyilia thoroldiana* communities and soil moisture and nutrient content in the Yellow River source watershed unit, while there was a significant negative correlation with soil conductivity and pH. Theses findings were consistent with findings of the study by Gao et al. [50] that fertile and humid soil habitats could promote the health and stability of plant communities. Further analyses showed significant associations between bacterial community characteristics and soil physicochemical properties and plant community characteristics, with significant (*p* < 0.05) or highly significant (*p* < 0.01) correlations between their diversity indices (Shannon’s index, number of OTUs, and Chao1 index) and soil physicochemical properties and plant community characteristics. This shows that the vegetation restoration of *Kengyilia thoroldiana* in the Yellow River source basin unit has realized the two-way promotion of soil fertility improvement and bacterial community optimization through the interaction mechanism of vegetation-soil-microorganism, and finally formed a self-reinforcing ecological virtuous circle [51].

Soil physical and chemical properties have a significant role in shaping the diversity and structure of the soil bacterial community in the rhizosphere of *Kengyilia thoroldiana* in the Yellow River source basin, and the influence mechanism of pH value is particularly prominent. The results showed that pH value regulated the rhizosphere soil bacterial community of *Kengyilia thoroldiana* through triple effects [52,53,54]: First, the reduction in pH value brings the soil close to neutrality, promotes the reproduction of beneficial bacteria such as rhizobia, and directly enhances bacterial diversity [52]. Second, a neutral pH environment can optimize cellulase and protease activities, improve carbon cycle efficiency, and indirectly enhance bacterial community diversity by increasing soil nutrients [53]. Third, soil neutralization promotes the recovery of plant communities, and the related symbiotic bacterial population expands accordingly, further enriching the bacterial community structure [54]. This process revealed that pH not only affected the microbial community in *Kengyilia thoroldiana* rhizosphere soil through soil fertility, but also regulated the stability of the bacterial community through plant–microbial interaction network feedback. In the practice of ecological restoration, adjusting the soil pH to a neutral range (6.5–7.5) can maximize the ecological service functions such as the carbon cycle and nutrient transformation driven by bacteria.

Future research will prioritize the establishment of a comprehensive “non-rhizosphere soil-rhizosphere soil” evaluation system, strengthening specificity analysis of rhizosphere effects through supplementary data collection from non-*Kengyilia thoroldiana* rhizosphere soils. Building upon this framework, metagenomic technologies will be integrated to deepen theoretical understanding of plant–soil–microbe interactions, while expanding habitat sampling scope to validate 16S rRNA gene-based functional predictions and explore unrecognized functional potentials of soil microorganisms. By employing structural equation modeling (SEM), we quantified the direct and indirect contributions of habitat factors—including plant community characteristics, soil physicochemical properties, and climatic gradients—to microbial community composition. This approach was complemented by network analysis to identify keystone species and their underlying driving mechanisms. Ultimately, this research establishes a closed-loop research framework spanning from foundational mechanistic elucidation to practical application translation, systematically advancing the in-depth analysis of rhizosphere effects and microbial–plant–environment interaction mechanisms. The framework provides scientific theoretical support for optimizing agricultural ecosystems and facilitating environmental restoration.

## 5. Conclusions

(1) Among the five topographic habitats, the water content and nutrient indicators such as organic carbon, total nitrogen, total phosphorus, and total potassium in the *Kengyilia thoroldiana* rhizosphere soil of the depression and transition zone were significantly higher than those of the sunny and shady slopes. Correspondingly, their community coverage, biomass, and plant height also performed better.
(2) The rhizosphere bacterial community of *Kengyilia thoroldiana* showed a differentiated response to five types of topographic habitats: the number of endemic bacteria in shady slope habitats was the highest, and the transitional zone habitats were the most abundant. The analysis of network topology shows that the bacterial community in shady slope and transitional zone habitats has a more complex co-occurrence network structure, and the number of network edges is significantly higher than that in other topographic habitats.
(3) Based on the hierarchical clustering distance, the rhizosphere soil bacterial community of *Kengyilia thoroldiana* was divided into two groups: habitat H3 was clustered into one group (group 1), and the other four habitats were classified into another group (group 2). The function prediction showed that the metabolic pathways of heterotrophic chemical energy and heterotrophic aerobic energy were dominant in all habitats, especially in depressions, mountain passes, and transitional zones, with relative abundance exceeding 93% (standardized value > 9300).
(4) Redundancy analysis (RDA) revealed that pH value and organic carbon were the core environmental factors driving the succession of soil bacterial communities in the rhizosphere of *Kengyilia thoroldiana* in five types of topographic habitats in the Yellow River source basin unit, and that they not only directly promoted bacterial diversity, but also indirectly affected bacterial metabolic function by regulating soil nutrient content.

## Figures and Tables

**Table 1:** Variations in rhizosphere soil characteristics and vegetation community traits of *Kengyilia thoroldiana* across different topographic habitats.

Habitat Samples	Coverage /(%)	Height /(cm)	Biomass /(g·m^−2^)	SWC /(%)	SEC /(μs·cm^−1^)	pH	SOC /(g·kg^−1^)	TN /(g·kg^−1^)	TP /(g·kg^−1^)	TK /(g·kg^−1^)
	*n* = 60	*n* = 60	*n* = 60	*n* = 3	*n* = 60	*n* = 60	*n* = 3	*n* = 3	*n* = 3	*n* = 3
H1	35.11 ± 3.9 c	18.63 ± 2.34 d	346.21 ± 20.97 d	23.13 ± 3.34 c	1098.36 ± 63.67 a	8.66 ± 0.05 a	5.60 ± 0.46 d	0.77 ± 0.16 d	0.32 ± 0.02 b	19.08 ± 2.12 c
H2	70.81 ± 6.82 a	34.87 ± 1.65 a	652.49 ± 20.65 a	40.38 ± 4.16 a	599.41 ± 38.50 c	7.97 ± 0.12 c	15.30 ± 1.53 a	1.55 ± 0.07 a	0.58 ± 0.06 a	25.28 ± 0.82 a
H3	44.05 ± 2.69 c	24.18 ± 1.60 c	395.31 ± 30.94 c	27.34 ± 2.85 c	932.54 ± 120.11 b	8.35 ± 0.02 b	5.84 ± 0.25 d	1.06 ± 0.14 b	0.36 ± 0.05 b	20.00 ± 0.46 bc
H4	56.12 ± 6.14 b	28.16 ± 2.07 b	468.17 ± 37.23 b	27.83 ± 1.27 c	832.02 ± 80.23 b	8.23 ± 0.03 b	8.43 ± 0.37 c	0.82 ± 0.02 d	0.39 ± 0.03 b	20.01 ± 1.21 bc
H5	70.25 ± 4.29 a	31.69 ± 1.36 a	623.71 ± 10.64 a	35.43 ± 3.23 b	648.72 ± 62.92 c	8.08 ± 0.06 c	10.75 ± 0.60 b	1.20 ± 0.08 b	0.52 ± 0.01 a	22.05 ± 1.41 b

Note: Within the same indicator, different lowercase letters indicate significant differences at *p* < 0.05 level. *n*: biological replicate number. SWC: soil water content, SEC: soil electrical conductivity, SOC: soil organic carbon, TN: soil total nitrogen, TP: soil total phosphorus, TK: soil total potassium. H1 is sunny slope, H2 is depression, H3 is shady slope, H4 is mountain pass, and H5 is transitional zone, the same below.

## Data Availability

Due to privacy reasons, the data is not publicly available. For further consultation, please contact the corresponding author.

## Supplementary Materials

The following supporting information can be downloaded at: https://www.mdpi.com/article/10.3390/microorganisms13112438/s1, Table S1. Unifactorial molecular network analysis of bacterial communities in the rhizosphere soil of *Kengyilia thoroldiana* across five habitats.
