# Supplementary material for: Bacterial Community Characteristics of Kengyilia thoroldiana Rhizosphere Soil in Different Topographic Habitats of the Yellow River Source Region and Their Response to Vegetation-Soil Factors
Source: Microorganisms. 2025 Oct 24;13(11):2438. doi: 10.3390/microorganisms13112438 (PMC12654118; doi:10.3390/microorganisms13112438)
Supplement: Supplementary file 1 [file microorganisms-13-02438-s001.zip › microorganisms-3853667-supplementary.pdf]

microorganisms

Supplementary Material

1 Supplementary Table

Supplementary Table S1. Unifactorial molecular network analysis of bacterial communities in the rhizosphere soil of *Kengyilia thoroldiana* across five habitats.

Habitat Samples	Number of nodes/coun	Number of edges/count	positive correlation/%
H1	50	426	56.57
H2	50	392	48.47
H3	50	618	50.16
H4	50	422	54.74
H5	50	548	49.45
